# A Novel Organic–Inorganic-Nanocomposite-Based Reduced Graphene Oxide as an Efficient Nanosensor for NO_2_ Detection

**DOI:** 10.3390/nano14241983

**Published:** 2024-12-11

**Authors:** Masoud Khaleghiabbasabadi, Hadi Taghavian, Pooya Gholami, Saeed Khodabakhshi, Mohammad Gheibi, Stanisław Wacławek, Miroslav Černík, Daniele Silvestri, Klaudia Barbara Raczak, Reza Moezzi

**Affiliations:** 1 Institute for Nanomaterials, Advanced Technology and Innovation, Technical University of Liberec, 46001 Liberec, Czech Republic; masoud.khaleghiabbasabadi@tul.cz (M.K.); hadi.taghavian@tul.cz (H.T.); mohammad.gheibi@tul.cz (M.G.); stanislaw.waclawek@tul.cz (S.W.); miroslav.cernik@tul.cz (M.Č.); barbara.klaudia.raczak@tul.cz (K.B.R.); 2 Faculty of Mechatronics, Informatics, and Interdisciplinary Studies, Technical University of Liberec, 46001 Liberec, Czech Republic; reza.moezzi@tul.cz; 3 Faculty of Chemical, Petroleum and Gas Eng, Semnan University, Semnan 35196, Iran; pooya1989gh@gmail.com; 4 Energy Safety Research Institute, Swansea University, Bay Campus, Swansea SA1 8EN, UK; saeidkhm@yahoo.com; 5 Association of Talent under Liberty in Technology (TULTECH), Sõpruse pst, 10615 Tallinn, Estonia

**Keywords:** toxic gas detection, nanosensor, reduced graphene oxide, nanocomposite, nitrous oxide (NO_2_)

## Abstract

There are three components to every environmental protection system: monitoring, estimation, and control. One of the main toxic gases with considerable effects on human health is NO_2_, which is released into the atmosphere by industrial activities and the transportation network. In the present research, a NO_2_ sensor is designed based on Fe_3_O_4_ piperidine-4-sulfonic acid grafted onto a reduced graphene oxide Fe_3_O_4_@rGO-N-(piperidine-4-SO_3_H) nanocomposite, due to the highly efficient detection of pollution in the air. In the first phase of the present study, the nanocomposite synthesis is performed in four steps. Afterward, the novel fabricated nanosensor is characterized through energy dispersive X-ray spectroscopy (EDX), Fourier transform infrared spectroscopy (FTIR), thermogravimetric analysis (TGA), Raman, surface area analysis, and field emission scanning electron microscopy (FE-SEM). To determine the optimal condition for sensor performance, graphene-based nanosensors are prepared with various weight percentages (wt%) of rGO-N-(piperidine-4-SO_3_H) (1 wt%, 5 wt%, 10 wt%, and 15 wt%). During the experimental process, the performance of the sensors, in terms of the sensitivity and response time, is investigated at different NO_2_ concentrations, between 2.5 and 50 ppm. The outputs of this study demonstrate that the synthesized nanosensor has the best efficiency at more than a 5 ppm contamination concentration and with at least 15 wt% of rGO-N-(piperidine-4-SO_3_H).

## 1. Introduction

Nitrogen oxide gases (NOx), like nitric oxide (NO) and nitrogen dioxide (NO_2_), can produce nitrous and nitric acids, which are toxic and harmful to humans and the environment and can cause photochemical pollution and acidic rain [1,2]. Generally, NOx gases are among the primary air pollutants produced during fuel combustion from both stationary and mobile sources, leading to increased efforts to control their emissions [3,4]. The burning of fossil fuels like coal, oil, and natural gas in power plants, transportation, and industrial activities significantly contributes to air pollution by emitting pollutants such as NOx, sulfur dioxide (SO_2_), and carbon monoxide (CO) [5]. Therefore, it is essential to develop durable and reliable NO_X_ gas sensors capable of providing real-time measurements to support public health and safety applications [6]. Nanomaterials have been used in various scientific and industrial sectors due to their extraordinary chemical and physical features [7,8,9,10]. In recent years, nanostructured carbon-based materials have garnered significant attention due to their potential applications across various fields, particularly in environmental protection and energy-related technologies [11,12,13]. Furthermore, nanostructured carbon-based materials have initiated wide applications for various gas-sensing technologies due to their fascinating chemical and physical properties [14,15,16], and they were found to be incredibly efficient as NO_2_ sensors [17,18].

Among nanostructured carbon-based materials, graphene derivatives, such as graphene oxide (GO) and reduced graphene oxide (rGO), offer unique properties that make them highly desirable for gas-sensing applications. These derivatives exhibit a substantial surface area, enabling enhanced gas adsorption and sensitivity, even towards low gas concentrations. Furthermore, their chemical reactivity allows for surface modification, facilitating selective gas sensing and minimizing interference from other gases. The exceptional electrical conductivity of graphene derivatives promotes efficient charge transfer, resulting in heightened sensitivity and quicker response times. Additionally, their robust mechanical strength and stability ensure durability when deployed in diverse environmental conditions. These materials can be fabricated in large quantities, seamlessly integrated into various sensor configurations, and are compatible with existing sensor technologies [19,20,21,22].

Nevertheless, the gas sensors based on only graphene do not show high performance and reliability due to a lack of desirable functional groups [23]. Therefore, to advance the performance of graphene-based sensors, other compounds, like polymers or metallic oxides, have been considered on the graphene surface, and the research showed enhancement of the selectivity and the sensitivity for desired gas sensors [24,25,26,27].

Herein, the current study presents the Fe_3_O_4_@rGO-N-(piperidine-4-SO_3_H) nanocomposite as a new NO_2_ sensor with high sensitivity and demanding a low response time even at low gas concentrations. The graphene surface is advantageous for the stability and dispersion of Fe_3_O_4_ nanoparticles (NPs) to attain small-size standardized NPs. Moreover, the graphene surface can act as a substratum to enhance migrating and capturing electrons from the nanocomposites’ conduction band [20,24].

Throughout the experimental procedure, the sensors’ sensitivity and response time are examined across various concentrations of NO_2_, ranging from 2.5 to 50 ppm. The outcomes of this investigation, such as the sensitivity, recovery, and response time, reveal that the fabricated nanosensor exhibits optimal performance at contamination concentrations exceeding 5 ppm and, notably, when incorporating a minimum of 15 wt% rGO-N-(piperidine-4-SO_3_H).

## 2. Materials and Methods

### 2.1. Fabrication of GO

GO was synthesized based on the modified Hummers method [28]. All chemicals, including natural flake graphite (325 mesh, 99.95%), H_2_O_2_ [30% (*w*/*w*) in H_2_O], ethanol, HCl, H_2_SO_4_ (98%), KMnO_4_, piperidine-4-sulfonic acid, FeCl_2_·4H_2_O, and FeCl_3_·6H_2_O, used for the syntheses in this investigation, were purchased from Sigma Aldrich Chemical Company (Rockville, MA, USA). The process was initiated after adding 2.5 g of graphite powder (325 mesh, 99%) in 1200 mL H_2_SO_4_ and gently mixing on a stirrer overnight at laboratory temperature. Afterward, 15 g of KMnO_4_ was stepwise added to the reaction while shaking before pouring it into the beaker, which had already been filled with 250 g of ice to the solution. Transformation was visually monitored by turning the light brown color to a bright yellow one after adding 280 mL of deionized water and 10% H_2_O_2_ (*v*/*v*). Then, the reaction mixture was centrifuged five times and washed well with 10% HCl (*v*/*v*). Finally, the GO nanoflakes were obtained after drying the mixture at 65 °C.

### 2.2. Synthesis of GO Functionalized with Piperidine-4-Sulfonic Acid [GO-N-(Piperidine-4-SO_3_H)]

A desired amount of GO was dispersed in H_2_O and ultrasonicated (the ultrasound eq. (2200 ETH-SONICA, Milano, Italy) for 30 min. Afterward, 0.1 g of piperidine-4-sulfonic acid was added to 62.5 mL of ethanol (EtOH). The reaction mixture was stirred for 24 h at 90 °C, and the resulting black mixture was filtered and washed several times with H_2_O/EtOH and dried at 70 °C for 24 h. Functionalizing GO with piperidine-4-sulfonic acid was performed through an epoxide group ring opening on the surface of GO and creating a C–N band [29].

### 2.3. Synthesis of Reduced Graphene Oxide-N-(Piperidine-4-SO_3_H) [rGO-N-(Piperidine-4-SO_3_H)]

Reduced GO nanoflakes were synthesized by adding NaBH_4_ as a reducing agent into the obtained GO-N-(piperidine-4-SO_3_H) suspension solution (360 mL, 2 g/L) at 80 °C for 1 h. The black nanoflake powders of rGO-N-(piperidine-4-SO_3_H) were obtained after filtration, washed with DI water, and dried at 70 °C [30].

### 2.4. Adding Fe_3_O_4_ Nanoparticles on rGO-N-(Piperidine-4-SO_3_H)

The preparation of Fe_3_O_4_ nanoparticles on rGO-N-(piperidine-4-SO_3_H) was stepwise carried out through the simultaneous dual-precipitation of FeCl_2_·4H_2_O and FeCl_3_·6H_2_O upon rGO-N-(piperidine-4-SO_3_H) to constitute Fe_3_O_4_@rGO-N-(piperidine-4-SO_3_H). First, the solution of FeCl_2_·4H_2_O and FeCl_3_·6H_2_O was mixed at a ratio of 1:2 [31]. It is 200 mg of FeCl_3_·6H_2_O and 125 mg of FeCl_2_·4H_2_O, which were simultaneously added to the deionized water (solution nr. 1, volume 50 mL). Second, the mixture of 10 mg/mL of rGO-N-(piperidine-4-SO_3_H) in deionized water was ultrasonicated (2200 ETH-SONICA ultrasound equipment, frequency: 45 kHz) for 30 min (solution nr. 2). Thirdly, the mixture solution’s pH and temperature were raised to 11.0 (through the addition of 30% *v*/*v* NH_3_:H_2_O) and 70 °C, respectively. The reaction mixture was left for 30 min on a stirrer at an adjusted temperature and pH. Then, the resultant mixture was left at laboratory temperature to cool down. Eventually, the resultant black powders of Fe_3_O_4_@rGO-N-(piperidine-4-SO_3_H) were centrifuged (4000 rpm/20 min), gently washed (6 times/DI water), and dried at 70 °C [31,32]. Table 1 distinguishes the specimens based on the rGO-N-(piperidine-4-SO_3_H) loadings.

### 2.5. Characterization

The structure of the synthesized nanosensor was characterized using various methods comprising Fourier transform infrared spectroscopy (FTIR), Raman, energy dispersive X-ray spectroscopy (EDX), thermo gravimetric analysis (TGA), and field emission scanning electron microscopy (FE-SEM). The FTIR spectrometer Nicolet iZ10 (Thermo Scientific, Evanston, IL, USA) was utilized to investigate the chemical bonds in the resulting samples. Raman analysis was performed with a Raman microscope DXR (Thermo Scientific, USA).

EDX analysis was conducted with a VG ESCALAB-200R spectrometer (Thermo Scientific, Evanston, IL, USA) equipped with a hemispherical electron analyzer for energy dispersion-based X-ray detection. For thermogravimetric analysis (TGA), a thermogravimetric analyzer Q500 (TA Instruments, New Castle, DE, USA) instrument was utilized under a nitrogen flow, covering temperatures ranging from 0 to 800 °C. Field emission scanning electron microscopy (FE-SEM) images were captured employing a Tescan Mira-3 Field Emission Gun Scanning Electron Microscope (Brno – Kohoutovice, Czech Republic) (FEG-SEM). The Brunauer–Emmett–Teller (BET) analysis was performed using a Micromeritics 3Flex Version 5.01 surface analyzer (Norcross, GA 30093, USA). The specific surface area, along with pore volume and size details, were extracted from data gathered during nitrogen-adsorption tests conducted at 77 K. Surface area calculations were executed using the BET technique.

### 2.6. Nanosensor Fabrication

The nanosensor fabrication was performed by employing electrodeposition and deposition techniques. First, the typical sputtering method was carried out to electrodeposit a layer of gold particles with a thickness of 300 nm on an alumina substrate. The spin-coating machine (Spin-1200D, MIDAS SYSTEM, Gyeonggido, Republic of Korea) has been utilized to deposit 0.2 g of the synthesized Fe_3_O_4_@rGO-N-(piperidine-4-SO_3_H) on the alumina after ultrasonication in 3 mL of EtOH. Then, the alumina substrate was put in the container, linked by silver paste, warmed for 2 h, and dried at 100 °C.

### 2.7. Gas Sensing Apparatus

**Figure 1:**
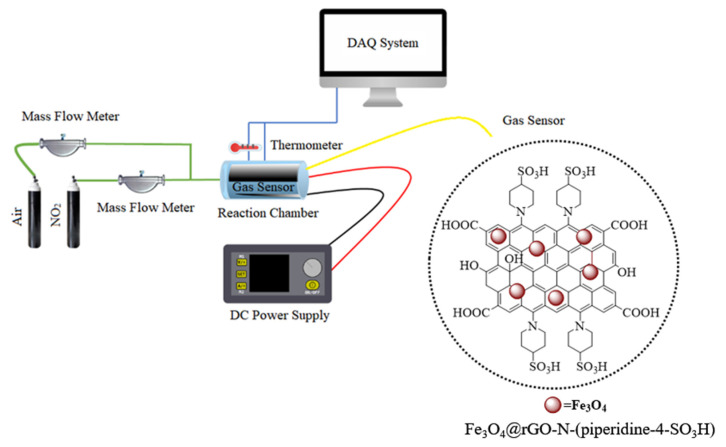
Scheme of the experimental gas-detection setup.

The gas sensing apparatus containing distinctive segments, which were elaborately connected, was utilized to detect the gases according to the schematic design shown in Figure 1 [15,16]. Dry air and the N_2_/NO_2_ mixture, as experimental gases, were supplemented from the gas tank compartments into the specific chamber. The chamber was equipped with a U-shaped glass container with a radius of 1.25 cm and 150 cm in length and a jacket heater to adjust the temperature during the process. In addition, a DC power supply was also connected to the system to monitor the output DC voltage. The produced sensor (including either GO or Fe_3_O_4_@rGO-N-(piperidine-4-SO_3_H) was placed in the U-shape quartz glass reactor attached to the measurement unit by cables. After the gas emission at the beginning of the test, the sensor’s complex started to collect the change in DC electrical conductance of its sensitive plates vs. time. The volt-amperometry technique (two-pole), aided by another multimeter, was utilized to assess the DC voltage. Mass flow controllers harness the overall flow rate of NO_2_ and dried air, as its carrier, during emission at a constant amount set in a range of 2.5–50 ppm. The reference resistance at the beginning of the measurement was assessed with dry air (R_air_). The ratio of the electrical resistivity measured based on NO_2_ gas and dry air is the main principle of evaluating the synthesized nanosensors’ sensitivity.

### 2.8. Nanosensor Performance

The Fe_3_O_4_@rGO-N-(piperidine-4-SO_3_H)-sensing test initiates after passing air through the chamber and keeping the pressure constant until the conductivity turns to the constant value shown by the detector. Then, the NO_2_ gas with a concentration increasing from 2.5 to 50 ppm is released into the testing chamber. The reduction reaction between the NO_2_ gas and the anions leads to the consumption of free electrons on the surface of the nanosensor, which is connected to the multimeter. The electrical resistivity increment, which is assessed using the volt-amperometry technique (two-pole), is attributed to the NO_2_ concentration. Although the sensitivity of the sensor increases by increasing the temperature; this study aims to gain the maximum sensitivity of the nanosensor at an ambient temperature; ergo, all experiments were performed in the laboratory without externally applied heat. The nanosensor system was recovered after the experiment through the injection of air into the chamber to remove the adsorbed gas and ions on the sensor’s surface and substitute them through the adoption of air.

### 2.9. Sensitivity Assessment

The calculation of the nanosensor sensitivity (S) is based on Equation (1):

(1) $$S=\frac{∆R}{R}\times 100\%$$

where R is the original signal amplitude, ∆R is the nanosensor signal change range, in a mix of gas with dried air, and the sensor resistance in pure dried air, respectively [15].

## 3. Result and Discussion

### 3.1. Characterization of Fe_3_O_4_@rGO-N-(Piperidine-4-SO_3_H)

**Figure 2:**
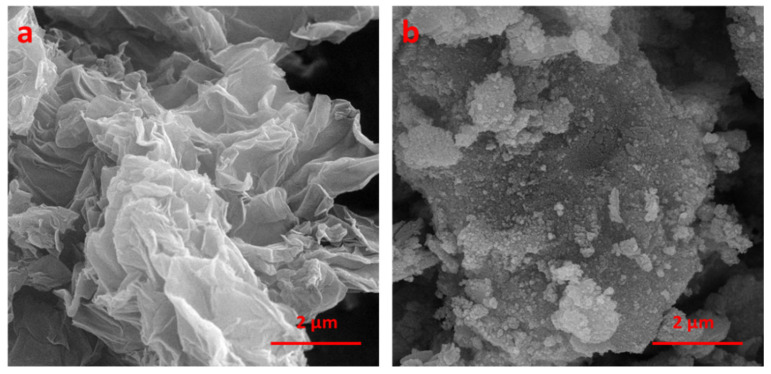
(**a**) FE-SEM of GO and (**b**) Fe_3_O_4_@rGO-N-(piperidine-4-SO_3_H); Sample D-15 wt%.

FE-SEM images were used to measure the size and morphology of GO and obtained Fe_3_O_4_@rGO-N-(piperidine-4-SO_3_H) (Figure 2). FE-SEM images reveal the random accumulation and folded thin flake morphology for both GO and Fe_3_O_4_@rGO-N-(piperidine-4-SO_3_H) specimens. It appears evident that Fe_3_O_4_ nanoparticles constitute the nanometric spherical shape statute on the rGO-N-(piperidine-4-SO_3_H). The average diameter varies from 25 to 100 nm.

**Figure 3:**
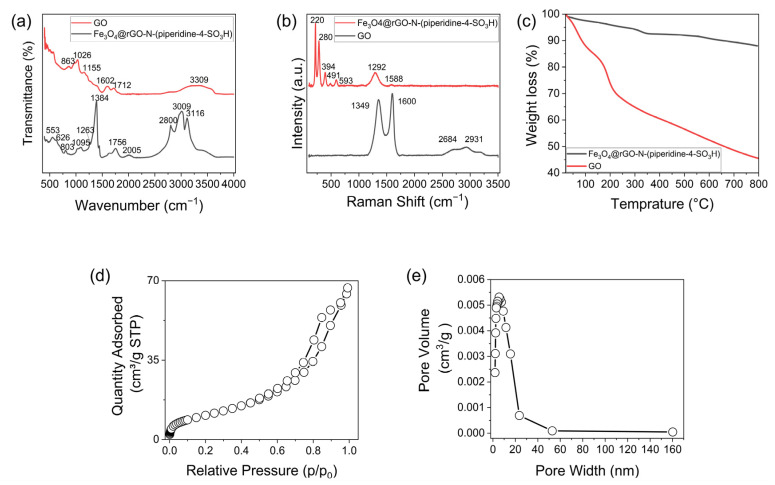
(**a**) FT-IR analysis of GO and Fe_3_O_4_@rGO-N-(piperidine-4-SO_3_H), (**b**) Raman curves of GO and Fe_3_O_4_@rGO-N-(piperidine-4-SO_3_H), and (**c**) TGA curves of GO and Fe_3_O_4_@rGO-N-(piperidine-4-SO_3_H); (**d**) BET surface area and (**e**) pore size distribution curves of Fe_3_O_4_@rGO-N-(piperidine-4-SO_3_H).

The FT-IR spectra of the GO and Fe_3_O_4_@rGO-N-(piperidine-4-SO_3_H) are illustrated in Figure 3a. The signal at 3309, 1712, 1602, and 1026 cm^−1^ are attributed to the stretching vibrations of O-H, C=O, C=C, and C-O functionalities, respectively [33,34]. The broad peak at 3309 cm^−1^ can be ascribed to the O-H stretching vibration of adsorbed water and carboxylic acid [34].

As can be seen further in Figure 3a, two main bands at around 626 and 553 cm^−1^ correspond to the stretching vibration of Fe–O bonds of the Fe_3_O_4_@rGO-N-(piperidine-4-SO_3_H) specimen [35]. Additionally, the peaks at 1384 cm⁻¹ and 803 cm⁻¹, corresponding to the vibrations of O=S=O and S–O bonds, confirm the presence of –SO_3_H groups in Fe_3_O_4_@rGO-N-(piperidine-4-SO_3_H) [36].

The small peak at 1263 cm^−1^ in the FT-IR spectra of Fe_3_O_4_@rGO-N-(piperidine-4-SO_3_H) is related to the stretching vibration of the amino C-N bond made by the amination of the GO films with piperidine-4-sulfonic acid [29,30]. Moreover, the distinct stretching vibrations observed at 2800, and 3116 cm^−1^ in the spectra of Fe_3_O_4_@rGO-N-(piperidine-4-SO_3_H) correspond to the stretching vibrations of the -CH_2_, and O-H bonds, respectively [37,38]. An evaluation of the FT-IR spectra confirms the successful functionalization of rGO with piperidine-4-sulfonic acid and Fe_3_O_4_ nanoparticles.

The Raman spectra of the GO and Fe_3_O_4_@rGO-N-(piperidine-4-SO_3_H) are demonstrated in Figure 3b. The D band and G band are visible at 1349 and 1600 cm^−1^ in the GO Raman spectra. The G bands of GO correlate to the first-order Raman scattering of the E_2g_ mode, whereas the D band is associated with edge defects and structural characteristics in GO. In the graphene lattice, structural defects, including vacancies and disorder, are linked to the D band. The graphene lattice’s sp^2^ carbon atoms’ vibration is represented by the G band. Oxygen-containing functional groups (epoxy, carboxyl, and hydroxyl groups) cause the sp^2^ carbon network to be disrupted in GO, which increases the Raman shift in the G band. Furthermore, additional defect structures might be responsible for the 2D and D+D’ bands seen in GO samples at 2684 and 2931 cm^−1^, respectively [39,40,41].

In addition, the Raman spectrum of Fe_3_O_4_@rGO-N-(piperidine-4-SO_3_H) demonstrates a characteristic peak of Fe_3_O_4_ at wavenumbers below 1000 cm^−1^, including 220, 280, 394, 491, and 593 cm^−1^ [42]. The observation that the G band (1588 cm^−1^) in the Raman spectrum of Fe_3_O_4_@rGO-N-(piperidine-4-SO_3_H) is shorter than that of GO further demonstrates the material’s successful fabrication. Additionally, a lower D band (1292 cm^−1^) is observed when compared to the GO sample due to the increased degree of disorder created by the magnetite particle formation on the surfaces of the rGO layers. The functionalization and magnetization of rGO using piperidine-4-sulfonic acid and Fe_3_O_4_ nanoparticles were all successfully accomplished, according to the Raman spectroscopy data [42,43].

Figure 3c shows the results of the thermo-gravimetric analysis of GO and Fe_3_O_4_@rGO-N-(piperidine-4-SO_3_H). As demonstrated in Figure 3c, GO has two main weight-loss steps [15,44]. The minor weight loss (14.6 wt% at up to 100 °C) is related to the evaporation of H_2_O molecules held on the layers of the GO [44]. Also, the second step (28.8 wt% at 250 °C) is primarily due to the degradation of functional groups on the surface of GO (carboxylic, hydroxyl, and epoxy groups) [45]. Thermal weight loss of the Fe_3_O_4_@rGO-N-(piperidine-4-SO_3_H) specimen is gradual, which can be distinguished by the main stages of 2.6 wt% of water evaporation (below 110 °C), 4.8 wt% due to the decomposition of the oxygen functional groups of the GO content (around 200 °C), and eventually, decomposition of the sulfonated groups (in the range of 400 °C to 600 °C) until the specimen lost 2 wt% of its initial weight [15]. The data regarding the BET analysis and pore volume of (Fe_3_O_4_@rGO-N-(piperidine-4-SO_3_H) are presented in Figure 3d,e. As illustrated in Figure 3, the specific surface area achieved for (Fe_3_O_4_@rGO-N-(piperidine-4-SO_3_H) was 40 m^2^/g. Additionally, the pore volume was determined as 0.00399 cm^3^/g for Fe_3_O_4_@rGO-N-(piperidine-4-SO_3_H). Moreover, the pore size of the synthesized nanocomposite was measured to be 9 nm.

**Figure 4:**
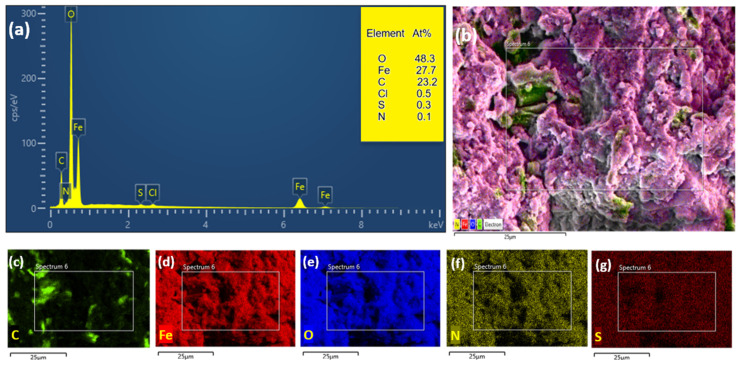
(**a**) EDX pattern of Fe_3_O_4_@rGO-N-(piperidine-4-SO_3_H), and (**b**) elemental mapping of the selected region of the SEM image of Fe_3_O_4_@rGO-N-(piperidine-4-SO_3_H) carbon (**c**), iron (**d**), oxygen (**e**), nitrogen (**f**), and sulfur (**g**).

EDX analysis was used to measure the elemental compositions of GO and Fe_3_O_4_@rGO-N-(piperidine-4-SO_3_H). The EDX images shown in Figure 4 clearly illustrate the expected chemical components for Fe_3_O_4_@rGO-N-(piperidine-4-SO_3_H) (C, O, S, Fe and N. The trace presence of Cl could be due to iron precursors)). In addition, Table 2 shows the C and O percentage amounts for GO and C, O, S, N, and Fe for Fe_3_O_4_@rGO-N-(piperidine-4-SO_3_H). An evaluation of the EDX results demonstrates no obvious impurities in the synthesized samples.

**Figure 5:**
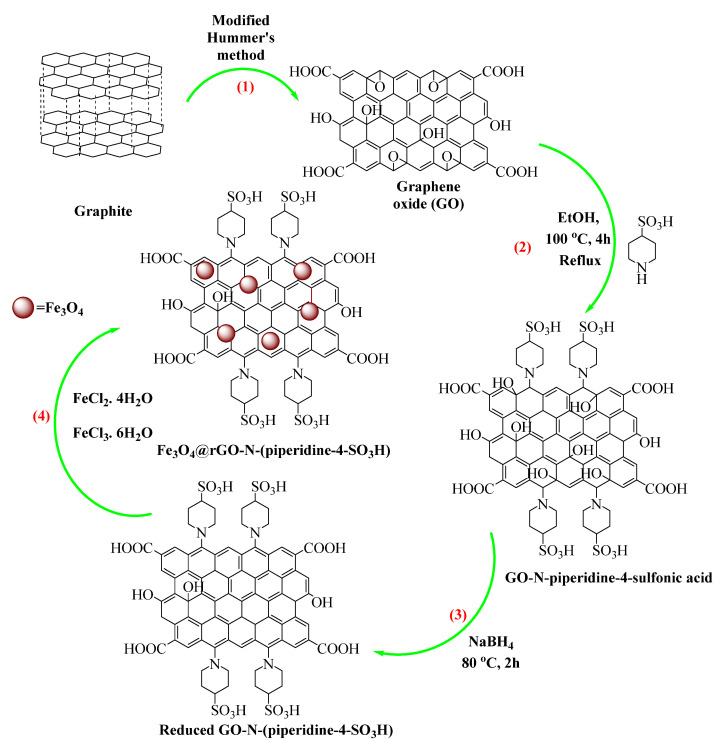
Schematic representation of Fe_3_O_4_@rGO-N-(piperidine-4-SO_3_H) synthesis. The numbers in the figure represent the phases and direction of the reaction.

The synthesis procedures of GO and Fe_3_O_4_@rGO-N-(piperidine-4-SO_3_H) were schematically proposed (Figure 5) according to the characterization studies using FT-IR, Raman, EDX, FE-SEM, and TGA. The process begins with graphite, which undergoes oxidation through a modified Hummer’s method to fabricate GO (step 1). The resulting GO is then functionalized with piperidine-4-sulfonic acid, incorporating nitrogen and sulfonic acid groups into the structure (step 2). Following this, the functionalized GO is reduced using sodium borohydride to form rGO-N-(piperidine-4-SO_3_H) (step 3). In the final step, Fe_3_O_4_ nanoparticles are combined with rGO-N-(piperidine-4-SO_3_H) for the synthesis of the Fe_3_O_4_@rGO-N-(piperidine-4-SO_3_H) composite (step 4), which is used as a sensor for detecting NO_2_ gas.

### 3.2. Fe_3_O_4_@rGO-N-(Piperidine-4-SO_3_H)-Sensing Mechanism

**Figure 6:**
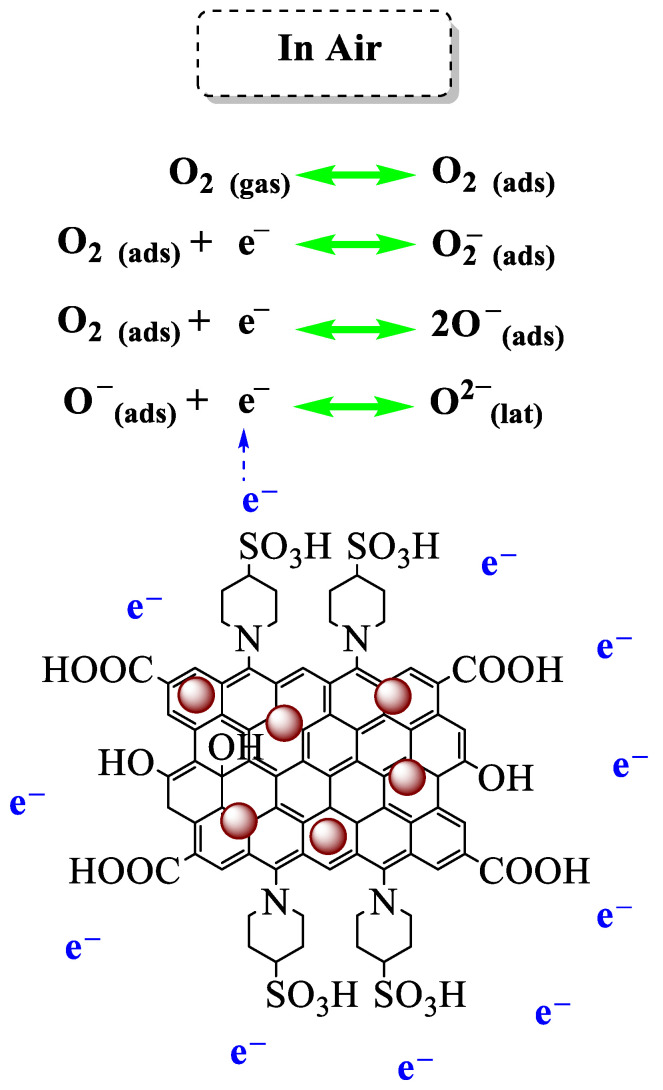
Adsorption mechanisms of the Fe_3_O_4_@rGO-N-(piperidine-4-SO_3_H) nanosensor.

The high surface-area-to-volume ratio of the synthesized nanosensor harnesses the conductivity through changes in surface charge [46]. The adsorption of any electronegative element on the surface of the nanosensor can contribute to the transfer of the charge and surface modification of the dielectric characteristic, and, eventually, change the conductivity of the nanosensor [15]. Accordingly, the sensing mechanism of the Fe_3_O_4_@rGO-N-(piperidine-4-SO_3_H) nanosensor is based on the adsorption of oxygen on the surface, capturing electrons and creating the ions of O_2_^−^, O^−^, and O^2−^ after exposure to air at the initial step of the test (Figure 6) [47,48].

$$O_{2}\left(gas\right)↔O_{2}\left(ads\right)$$

$$O_{2}\left(ads\right)+e↔O_{2}^{-}\left(ads\right)$$

$$O_{2}\left(ads\right)+e↔{2O}^{-}\left(ads\right)$$

$$O^{-}\left(ads\right)+e↔O^{2-}\left(lat\right)$$

**Figure 7:**
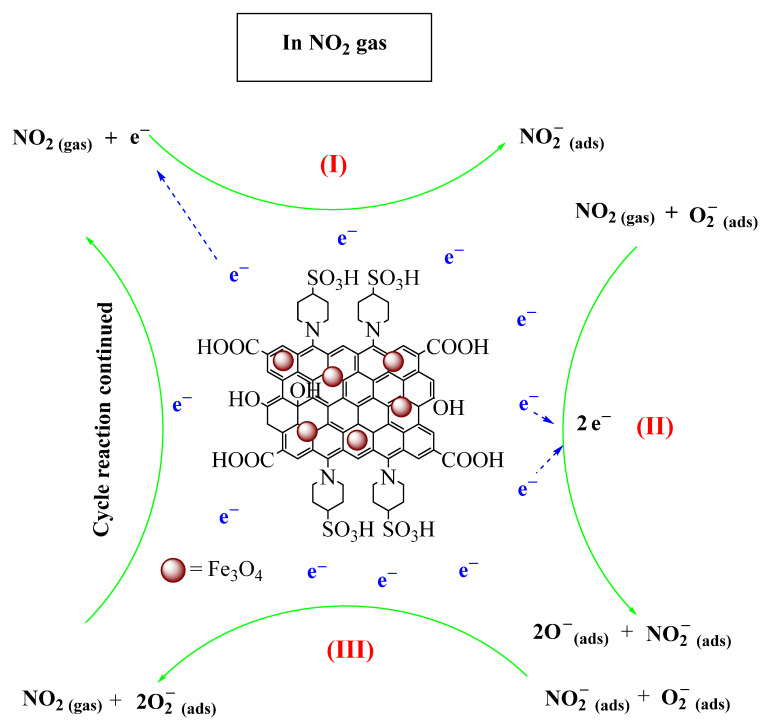
In NO_2_ gas reactions and Fe_3_O_4_@rGO-N-(piperidine-4-SO_3_H)-sensing mechanisms.

The characteristics of the active sites on the nanosensor’s surface govern the gas-sensing response [46]. Hereupon, the sulfonic acid group in the nanosensor acts as an intrinsically electron-withdrawing group, facilitating the adsorption of electrons from the electronegative O₂ in air [15]. The Fe_3_O_4_, as the metal oxide part of the gas sensor and is aided by the rGO, is the responsible site for the oxidation–reduction reactions among the absorbed oxide anions and NO_2_ gas [15]. The concentration of newly formed surface anions on the nanosensor after gas exposure alters its conductivity. Electron-donating gases, such as volatile organic compounds (VOCs), introduce additional electrons to the surface, increasing conductivity. Conversely, the electron-withdrawing nature of NO_2_ gas leads to its reduction, consuming oxide anions, and reducing conductivity (increasing resistivity). These mechanisms are schematically illustrated in Figure 7 [47,48].

(I)NO2gas+e−→NO2−(ads)

(II)NO2gas+O2−ads+2e−→NO2−ads+2O−ads

(III)NO2−ads+O2−ads→2O2−ads+NO2

Then, the cycle reaction continued from the reaction (I).

These reactions led to a decrease in the electron concentration on the surface of the sensors, which reduced its conductivity (increased its resistance). This change in conductivity allows for the detection of NO_2_ [48].

### 3.3. Fe_3_O_4_@rGO-N-(Piperidine-4-SO_3_H)-Sensing Performance

**Figure 8:**
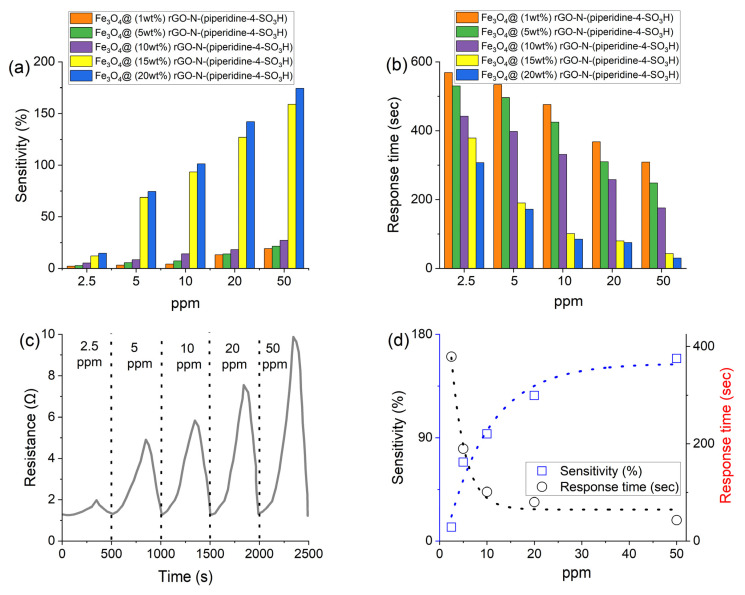
(**a**) Sensitivity for different samples of Fe_3_O_4_@rGO-N-(piperidine-4-SO_3_H) in several NO_2_ concentrations (2.5 and 50 ppm) at RT. (**b**) Response time for several samples of Fe_3_O_4_@rGO-N-(piperidine-4-SO_3_H) (X = rGO-N-(piperidine-4-SO_3_H) X = 1 wt%, 5 wt%, 10 wt%, 15, and 20 wt%) in a variety of NO_2_ concentrations at RT. (**c**) The relationship between resistance and time at RT cyclical curves used for (sample D) at various NO_2_ concentrations. (**d**) Sensitivity for (sample D) at RT for 5 ppm NO_2_.

A gas-sensing evaluation of the Fe_3_O_4_@rGO-N-(piperidine-4-SO_3_H) nanosensors shows the dependency of the sensor’s sensitivity to the gas concentrations and active site concentration. Figure 8a illustrates the correlation among various Fe_3_O_4_@rGO-N-(piperidine-4-SO_3_H) samples (1–20%) and different NO_2_ gas concentrations (2.5 ppm–50 ppm). Based on the expectation, increasing the gas concentrations strengthens the sensitivity of the nanosensor. The emission of more electron-withdrawing NO_2_ gas contributes to the consumption of more anions on the sensor’s surface and abruptly changes the resistivity. However, the percentage of the active sites on the nanosensor’s surface holds the dominant effect on the sensitivity performance. Increasing the active sites from 10 wt% to 15 wt% was a real game changer in increasing the sensitivity of the sensing system (Figure 8a), even though all samples showed a slight impact on sensitivity at the NO_2_ concentration lower than 10 ppm.

The dependency of the response time of the gas sensing to the NO_2_ concentration and the nanosensor’s active sites is illustrated in Figure 8b. Increasing the active site number and gas concentration from 1% to 20% and 2.5 ppm to 50 ppm, respectively, decreased the response time of the system significantly. Overall, NO_2_ is an electron-withdrawing gas, ready to be adsorbed on electron-rich sites, such as free electron pairs of S or O atoms in SO_3_^−^. Therefore, a higher NO_2_ concentration and active site n to more redox reactions and shorter response times of the gas-sensing system. Based on the results of the sensor’s sensitivity, increasing the active site number to over 15 wt% leads to a significant acceleration of the redox reactions in the nanosensor’s surface and a decrease in the response time. Graphene-based nanosensors are prepared with varying weight percentages of rGO-N-(piperidine-4-SO_3_H) (1 wt%, 5 wt%, 10 wt%, and 15 wt%) to determine the optimal sensor performance conditions. Therefore, the results show that the synthesized nanosensor performs most efficiently at contamination levels above 5 ppm and with a minimum of a 15 wt% rGO-N-(piperidine-4-SO_3_H) content.

Figure 8c displays the results of NO_2_ detection at different concentrations ranging from 2.5 ppm to 50 ppm, the relationship between resistance and time at room temperature (RT). The curve indicates a sudden shift upon exposure to the gas, and the same pattern is observed when the gas is cleared and replaced with fresh air.

The nanosensor was prepared by treating the active sites with air, resulting in different numbers of oxygen groups on the graphene surface. The resistance levels exhibited a decrease after the air was injected into the system. Upon NO_2_ emissions into the chamber, followed by electron-withdrawing from the adsorbed oxygen on the nanosensor’s surface, the initial resistance was recorded after the first air injection (1.8 KΩ) and underwent a decline from 1.8 KΩ to 1.5 KΩ in 2.5 ppm of NO_2_. This mechanism was repeated similarly for other NO_2_ concentrations.

The sensing accuracy of the nanosensor depends on the concentration of NO_2_ gas, in a way that decreasing the NO_2_ concentration leads to a reduction in sensing performance. Figure 8d illustrates the response time and sensitivity of the nanosensor based on alterations in the NO_2_ concentration. Accordingly, the sensitivity of the system increases as the NO_2_ concentration increases. As shown in Figure 8d, there is a direct relationship between the sensitivity and NO_2_ concentration, where increasing the NO_2_ concentration from 2.5 to 50 ppm enhances the sensitivity (S) from 12% to 159%.

Table 3 shows the experimental data of the nanosensor (sample D) at various concentrations of NO_2_ at room temperature. As can be seen in Table 3, the best response time for NO_2_ detection occurred at a concentration of 50 ppm, while the lowest response was obtained at concentrations of NO_2_ between 2.5 and 5 ppm. The higher reactivity of O_2_ gas compared to NO_2_ likely played a significant role in improving the response, as shown in entries 1–2.

A comparison between the outcomes of the current work and described research is visible in Table 4. The outcomes demonstrate that the recognition variety, responsiveness, and optimum temperature of the Fe_3_O_4_@rGO-N-(piperidine-4-SO_3_H) sensor were improved compared to other research. These outcomes indicate that the chemical treatment of reduced GO beside the SO_3_H group and the magnetization of rGO with Fe_3_O_4_ enhanced the performances of graphene-based NO_2_ sensors. In addition, the presence of amine and sulfonic acid functional groups on the surface of Fe_3_O_4_@rGO-N-(piperidine-4-SO_3_H) could increase the possible interaction between the sensor and some gases, such as H_2_S [30,49].

## 4. Conclusions

The present study described the fabrication, characterization, and application of the Fe_3_O_4_@rGO-N-(piperidine-4-SO_3_H) nanocomposite to detect NO_2_ gas with high selectivity and sensitivity. The sensing capability of the synthesized Fe_3_O_4_@rGO-N-(piperidine-4-SO_3_H) nanosensor is based on the high electronegative oxygen adsorption on the nanosensor’s surface, electron capture, and redox reactions between the target gases on the nanosensor surface, which alter the conductivity of the system (change in the resistivity). The Fe_3_O_4_@rGO-N-(piperidine-4-SO_3_H) sensor can spontaneously recover to its initial state through air flow without requiring thermal or chemical assistance. The experimental process illustrates that the nanosensor showed the best efficiency at more than a 5 ppm NO_2_ concentration and a minimum of 15 wt% graphene-based content in the nanocomposite structure. Meanwhile, at more than 10 ppm of NO_2_, the sensitivity and response time are measured more than 100% and less than 100 s, respectively. The results indicate that the sensor’s response time and detection range have been improved compared to previous studies. This research demonstrates that modifying GO with piperidine sulfonic acid on GO sheets is an effective novel nanosensor to enhance its gas-sensing capabilities.

## Figures and Tables

**Table 1:** Samples with different rGO-N-(piperidine-4-SO_3_H) loadings.

Samples	Content
A	Fe_3_O_4_@ (1 wt%) rGO-N-(piperidine-4-SO_3_H)
B	Fe_3_O_4_@ (5 wt%) rGO-N-(piperidine-4-SO_3_H)
C	Fe_3_O_4_@ (10 wt%) rGO-N-(piperidine-4-SO_3_H)
D	Fe_3_O_4_@ (15 wt%) rGO-N-(piperidine-4-SO_3_H)
E	Fe_3_O_4_@ (20 wt%) rGO-N-(piperidine-4-SO_3_H)

**Table 2:** EDX quantitative elemental analysis of GO and Fe_3_O_4_@rGO-N-(piperidine-4-SO_3_H).

Samples	Elements	C	O	S	N	Fe
GO	Weight %	46.4	53.6	_	_	_
	Atomic %	53.5	46.4	_	_	_
Fe_3_O_4_@rGO-N-(piperidine-4-SO_3_H)	Weight %	10.6	29.4	0.3	0.1	58.9
	Atomic %	23.1	48.2	0.3	0.1	27.7

**Table 3:** The various experimental values of the nanosensor (sample D) at RT for several NO_2_ concentrations.

No.	NO_2_ Concentration	The Percentage of Response	Response Time (s)	Recovery Time (s)
1	2.5	12	379	640
2	5	68.8	190	415
3	10	93.4	101	174
4	20	126.9	80	112
5	50	158.9	43	95

**Table 4:** Comparison of NO_2_-sensor capability from the current work and previous research for other materials utilized in experimental-based NO_2_-sensing procedures.

Samples	NO_2_ (ppm)	The Percentage of Response	Temperature (Celsius)	Response Time (s)	Fabrication Method	Recovery Time (s)	References
rGO	4.5	20	Room temperature	>300	Electrospinning and electrostatic self-assembly	>300	[50]
rGO nanofibrous mesh fabric	1	13.6	Room temperature	~1500	Electrospinning	~3000	[51]
rGO-Au	5	1.3	50	132	Hydrothermal	386	[52]
rGO	5	30	Room temperature	>10	Spin-coating of onto a mounted sensor chip	600	[53]
ZnO-decorated rGO fibers	1.5	1.4	Room temperature	405	Wet spinning of rGO fibers and hydrothermal treatment	760	[54]
ZnO- rGO	5	25.6	Room temperature	165/499	Dip-coating	499	[25]
Urc-ZGO	100	17.4	Room temperature	780	One step solvothermal method	1980	[55]
rGO-WO_3_	5	769	Room temperature	540	One-pot polyol process combined with MOD	1080	[56]
SnS_2_-rGO	11.5	56.8	80	360	Chemical method and thermal reduction	3180	[57]
MoS_2_-rGO	3	1.2	160	8	Two step wet chemical method	20	[58]
RGO-In_2_O_3_	30	8.2	Room temperature	240	Hydrothermal	1440	[59]
Fe_3_O_4_@rGO-N-(piperidine-4-SO_3_H)	5	68.8	Room temperature	190	Electrodeposition	415	Present work

## Data Availability

The data for this study are available from the corresponding author upon reasonable request.
